# Osteogenic differentiation of mesenchymal stromal cells in two-dimensional and three-dimensional cultures without animal serum

**DOI:** 10.1186/s13287-015-0162-6

**Published:** 2015-09-07

**Authors:** Eeva Castrén, Tarvo Sillat, Sofia Oja, Ariel Noro, Anita Laitinen, Yrjö T Konttinen, Petri Lehenkari, Mika Hukkanen, Matti Korhonen

**Affiliations:** Institute of Biomedicine, Anatomy, Biomedicum Helsinki, University of Helsinki, PO Box 63, Helsinki, Finland; Department of Medicine, Helsinki University Central Hospital, PO 700, 00029 HUS Helsinki, Finland; Division of Hemato-Oncology and Stem cell Transplantation, Hospital of Children and Adolescents, Helsinki University Central Hospital, Helsinki, Finland; Finnish Red Cross Blood service, Kivihaantie 7, 00310 Helsinki, Finland; ORTON Orthopaedic Hospital of the Invalid Foundation, PO 29, 00281 Helsinki, Finland; Departments of Anatomy and Surgery, University of Oulu, Aapistie 7, 90220, Oulu, Finland

## Abstract

**Introduction:**

Bone marrow-derived mesenchymal stromal cells (MSCs) have been intensely studied for the purpose of developing solutions for clinical tissue engineering. Autologous MSCs can potentially be used to replace tissue defects, but the procedure also carries risks such as immunization and xenogeneic infection. Replacement of the commonly used fetal calf serum (FCS) with human platelet lysate and plasma (PLP) to support cell growth may reduce some of these risks. Altered media could, however, influence stem cell differentiation and we address this experimentally.

**Methods:**

We examined human MSC differentiation into the osteoblast lineage using in vitro two- and three-dimensional cultures with PLP or FCS as cell culture medium supplements. Differentiation was followed by quantitative polymerase chain reaction, and alkaline phosphatase activity, matrix formation and matrix calcium content were quantified.

**Results:**

Three-dimensional culture, where human MSCs were grown on collagen sponges, markedly stimulated osteoblast differentiation; a fourfold increase in calcium deposition could be observed in both PLP and FCS groups. PLP-grown cells showed robust osteogenic differentiation both in two- and three-dimensional MSC cultures. The calcium content of the matrix in the two-dimensional PLP group at day 14 was 2.2-fold higher in comparison to the FCS group (*p* < 0.0001), and at day 21 it was still 1.3-fold higher (*p* < 0.001), suggesting earlier calcium accumulation to the matrix in the PLP group. This was supported by stronger Alizarin Red staining in the PLP group at day 14. In two-dimesional PLP cultures, cellular proliferation appeared to decrease during later stages of differentiation, while in the FCS group the number of cells increased throughout the experiment. In three-dimensional experiments, the PLP and FCS groups behaved more congruently, except for the alkaline phosphatase activity and mRNA levels which were markedly increased by PLP.

**Conclusions:**

Human PLP was at least equal to FCS in supporting osteogenic differentiation of human MSCs in two- and three-dimensional conditions; however, proliferation was inferior. As PLP is free of animal components, and thus represents reduced risk for xenogeneic infection, its use for human MSC-induced bone repair in the clinic by the three-dimensional live implants presented here appears a promising therapy option.

**Electronic supplementary material:**

The online version of this article (doi:10.1186/s13287-015-0162-6) contains supplementary material, which is available to authorized users.

## Introduction

Mesenchymal stromal cells (MSCs) are multipotent progenitor cells that can be isolated from bone marrow, adipose tissue, and other mesenchymal tissues. They can be differentiated into bone, fat, cartilage, tendon, and muscle [1]. These properties make MSCs an attractive agent for regenerative medicine and especially for reconstructive surgery in repair of bone defects [2–4]. MSCs also possess immunosuppressive properties and have been used for the treatment of acute graft versus host disease [5]. However, the risks involved in cell expansion for clinical bone repair still require careful evaluation. A number of preliminary studies indicate that local and systemic administration of autologous MSCs is safe [6–8]. To date, no serious complications such as tumorigenesis have been reported [9]. Fetal calf serum (FCS) has been widely used in osteogenic MSC cultures. Its xenogeneic origin poses two potential problems: the risk of xenogeneic infections and the risk of adverse immune reactions. Use of animal-derived cell culture materials contaminates both the cell surface and intracellular structures with xenoantigens [10, 11]. Exposure to these antigens may lead to immune reactions and graft rejection [12]. Use of animal-derived cell culture supplements introduces the risk of transferring bovine spongiform encephalopathy and other yet unknown zoonoses [13, 14]. Therefore, for clinical purposes, it is essential to find culture methods enabling osteogenic differentiation of MSCs that do not utilize animal-derived materials.

The combination of platelet lysate and plasma (PLP) supports the growth and proliferation of MSCs [15]. PLP is easily obtained from normal human blood. PLP contains growth factors, such as platelet-derived growth factor, transforming growth factors β1 and β2, insulin-like growth factor, epidermal growth factor, and endothelial cell growth factor, which all support bone healing. This makes PLP a promising material for human osteogenic MSC cultures [16]. However, it is uncertain whether PLP-grown MSCs display osteogenic potential comparable to cells grown using the standard FCS-based culture media [17–19].

It is envisioned that the repair of bone defects requires the implantation of the therapeutic cells into patients within three-dimensional (3D) cell matrices. The matrix must both support the growth of MSCs and the differentiation of the osteoblast phenotype, and it must be biocompatible with bone healing [20]. Several different scaffold materials have been employed in animal models [21–23]. In our study, we have used a collagen sponge scaffold (Spongostan®; Ferrosan, Soeborg, Denmark) which is already in clinical use for surgical hemostasis. It has previously been used as a cell culture scaffold to repair bone defects in congenital pseudoarthrosis [7] and as a 3D scaffold for autologous chondrocyte transplantation [24].

In this paper, we have compared the osteogenic differentiation potential of human MSCs that have been grown in FCS and PLP. After establishing the culture methods in a two-dimensional (2D) cell culture system, we plated the cells into Spongostan collagen matrix. We used several different assays to follow the osteogenic differentiation and to compare the FCS and PLP cultures against each other in both 2D and 3D cultures. We demonstrate that MSCs cultured without animal-derived components are capable of osteogenic differentiation and have thus clinical potential for bone repair.

## Methods

### Cell culture

Human MSCs were obtained from bone marrow aspirates from healthy volunteer donors aged 20–30 years after signed informed consent. The protocol was approved by the Ethics committee of the Helsinki University Central Hospital, Finland. Bone marrow (20 ml) was aspirated under local anesthesia from the posterior iliac crest into heparinized tubes. Mononuclear cells were isolated with a Ficoll-Paque (Pharmacia, Uppsala, Sweden) density gradient centrifugation and plated at the density of 4 × 10^5^ cells/cm^2^ in either PLP or FCS expansion medium. The cells were kept in either FCS or PLP medium throughout the experiments. After about 3 days of culture, the plates were washed thoroughly with phosphate-buffered saline (PBS) to remove nonadherent cells. The medium was changed every 3 to 4 days. After 3 weeks of culture, the primary cultures were passaged at 10^3^ cells/cm^2^. The cells were thereafter passaged always when they reached 80 % confluency. A standard panel of surface markers was used to characterize the cells (see Additional file 1). Differentiation assays were used to demonstrate adipogenic and osteogenic differentiation potential (see Additional file 1).

### Preparation of expansion media

The basic medium consisted of low-glucose Dulbecco’s modified Eagle’s medium supplemented with 100 U/ml penicillin, 100 μg/ml streptomycin (EuroClone, S.p.A. P.IVA, Italy) and 2 mM L-glutamine (EuroClone). FCS expansion medium was made from basic medium supplemented with 10 % FCS (tested for its ability to support MSC expansion; StemCell Technologies, Vancouver, BC, Canada).

PLP was obtained by pooling four platelet units containing approximately 300 × 10^9^ platelets from the local blood bank (Finnish Red Cross Blood Service, Helsinki, Finland). Platelets were centrifuged, suspended in AB plasma (Octaplas, Finnish Red Cross Blood Service) and frozen. The platelets underwent five rapid freeze–thaw cycles. Before use, the ability of the platelet lysate to support MSC expansion was tested using FCS expansion medium as a reference. The PLP expansion medium consisted of 2.5 % AB-plasma (Octaplas), 0.5 % platelet lysate (final concentration approximately 0.8 × 10^8^ platelets/ml) and 40 IU/ml heparin (Sigma, St. Louis, MO, USA) added to the basic medium.

### Osteogenic differentiation of MSCs

Osteogenic differentiation medium consisted of either FCS or PLP expansion medium supplemented with 10 mM β-glycerophosphate, 0.1 μM dexamethasone, and 200 μM ascorbic acid-2-phosphate (all from Sigma). FCS or PLP expansion medium alone without these supplements was used for control samples.

For 2D cultures, cells from passage two were plated at 5 × 10^3^/cm^2^ in expansion medium. When they reached 70 % confluence, they were changed into a differentiation medium, and cultured further up to 28 days.

For 3D cultures, 25 × 10^3^ cells from passage two in 75 μl expansion medium were pipetted into 7 × 7 × 10 mm Spongostan® collagen scaffold (Ferrosan) in 24-well plates. After an adhesion period of 6 hours, 1 ml expansion medium was added to each well. The following day, the cells were changed into differentiation medium and cultured up to 28 days.

The increase in cell number during the osteogenic differentiation was assessed by harvesting the 2D cultured cells with trypsin-EDTA and counting them with a Coulter cell counter. Cells cultured in the 3D system were harvested by dissolving the collagen scaffolds with collagenase B (1 mg/ml; Roche, Mannheim, Germany) and counted thereafter as above.

### Assays for osteogenic differentiation

To detect alkaline phosphatase (ALP) activity, we used the SensoLyte™ pNPP Alkaline Phosphatase Assay Kit Colorimetric (Anaspec, Fremont, CA, USA). Cells from passage three were plated in six-well plates (2D cultures) or in collagen scaffolds (3D) and grown up to 21 days. To recover the cells, the plates were washed twice and harvested in assay buffer with 1 % Tx-100. For 3D cultures, the collagen scaffolds were first dissolved with 1 mg/ml collagenase B, after which the cells were washed twice and suspended in assay buffer with 1 % Tx-100. The cell suspension was collected into Eppendorf tubes and incubated for 10 minutes at 4 °C under agitation. Aliquots of the extract were mixed with para-nitrophenyl phosphate (pNPP) substrate solution and the results were read spectrophotometrically at 405 nm.

To analyze the deposition of a collagenous and mineralized matrix in 2D cultures, the cells from passage one were plated in 24-well plates and cultured up to 21 days. The cell cultures were washed with PBS and fixed in 10 % paraformaldehyde (PFA) for 5 minutes at room temperature. In order to detect collagenous matrix deposition, the cell cultures were stained with 1 % Sirius Red (Direct Red 80; Sigma) in saturated picric acid for 1 hour, after which the plates were washed extensively with 0.5 % acetic acid. The plates were dried and scanned with the Epson Perfection V500 Photo Scanner (Epson Inc., Long Beach, CA, USA) and converted into binary images with the Adobe Photoshop CS3 Extended program (Adobe systems Inc., San Jose, CA, USA). The optical density (OD) of the Sirius Red-stained cultures was measured with the public domain ImageJ software (http://imagej.net/Welcome) and the values were calibrated according to the ImageJ instructions. To detect mineralized matrix, the matrix was stained with 2 % Alizarin Red S (Sigma) for 30 minutes after which the plates were washed extensively with tap water. The plates were dried and scanned and optical densities were measured as above.

Cells cultured in 3D matrices from passage three were fixed with 4 % PFA for 30 minutes, frozen in liquid nitrogen and mounted into Tissue-Tek OCT compound (Sakura Finetek, Torrance, CA, USA). Cryostat sections (6 μm) were stained with hematoxylin and eosin to visualize the general structure of the 3D matrix. Other sections were incubated with Alizarin Red for 30 minutes, washed thoroughly with tap water and photographed with Olympus Provis AX70 (Olympus, Tokyo, Japan).

In order to measure calcium deposition in the cell cultures from passage three, calcium was extracted in 40 μl (2D cultures) or 200 μl (3D cultures) 0.5 M HCl at 4 °C for 24 hours. After clearing the extracts by centrifugation, aliquots were mixed with an o-cresolphthalein complexone reagent (StanbioTotal Calcium LiquiColor, Boerne, TX, USA) according to the manufacturer’s instructions. The results were read spectrophotometrically at 550 nm. Standard curves were included in each measurement.

### Quantitative real-time polymerase chain reaction

For isolation of total RNA, the 3D cultures were first disrupted using an ultrasound homogenizer. RNA was isolated with TRIzol reagent (Invitrogen, Paisley, UK) according to the manufacturer’s instructions from both 2D and 3D cultured cells from passage three. First strand cDNA was synthesized from 200 ng total RNA using the Superscript enzyme (SuperScript® VILO™ cDNA Synthesis Kit; Invitrogen). cDNA synthesis without sample and without enzyme were used as negative controls. Quantitative real-time polymerase chain reaction (RT-PCR) was run in a LightCycler PCR machine using LightCycler FastStart DNA Master SYBR Green I kit (Roche) twice for each sample. Primers were designed with Primer3 (SourceForge, Roche, Mannheim, Germany), the sequences were searched using the National Center for Biotechnology Information (NCBI) Entrez search system, and sequence similarities using the NCBI Blastn program. Primer sequences used were 5′-GGTTTCAGTGGTTTGGATGG-3′ (forward) and 5′- ATTGGCACCTTTAGCACCAG-3′ (reverse) for collagen I α1 (COL1A1; producing amplicon length of 396 bp); 5′- TCAACACCAACGTGGCTAAG-3′ (forward) and 5′-CACAATGCCCACAGATTTCC-3′ (reverse) for ALP (356 bp); 5′-TCACACTCCTCGCCCTATTG-3′ (forward) and 5′- TCAGCCAACTCGTCACAGTC-3′ (reverse) for osteocalcin (OCN; 244 bp); 5′- TTACTGTCATGGCGGGTAAC-3′ (forward) and 5′- ATGCGCCCTAAATCACTGAG-3′ (reverse) for Runx2 (295 bp) and 5′-TCACCCACACTGTGCCCATCTACGA-3′(forward) and 5′-CAGCGGAACCGCTCATTGCCAATGG-3′ (reverse) for β-actin (295 bp).

To obtain the standard curve for quantitative PCR, the genes of interest were amplified in a PCR reaction, extracted from the agarose gel and cloned into a pCRII-TOPO vector (Invitrogen). After identification of the plasmid by restriction enzyme analysis and sequencing, its concentration was determined spectrophotometrically and serial dilutions were prepared for quantitative PCR analysis. The copy numbers of mRNA were determined in duplicate for each culture condition and normalized against 1 × 10^6^ copies of the housekeeping β-actin gene.

### Statistics

All differentiation experiments were carried out at least three times using cells from different donors. Data are expressed as mean ± standard deviation. Statistical significance was evaluated using a two-tailed Student’s *t*-test and <0.05 was considered significant.

## Results

### Characterization of MSCs

Cultured cells were shown to be MSCs by flow cytometry. Both PLP- and FCS-grown cells expressed commonly used MSC markers (CD73, CD90, and CD105), but were negative for CD14, CD19, CD45, HLA-DR (hematopoietic) and CD34 (hematopoietic/endothelial) markers (Figure S1 in Additional file 2) [25]. The cells were capable of osteogenic and adipogenic (Figure S2 in Additional file 2) differentiation.

### Osteogenic differentiation in 2D cultures

#### Increase in cell number during differentiation in 2D cultures

**Fig. 1:**
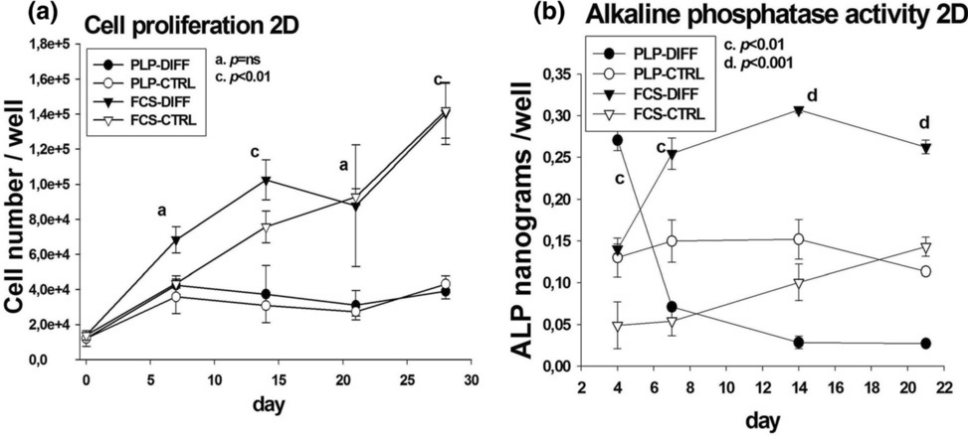
Cell proliferation and alkaline phosphatase (ALP) activity in two-dimensional (2D) cell culture. Cells were plated at the density of 5 × 10^3^/cm^2^, changed into osteogenic medium and cultured up to 28 days in 2D culture. **a** Cell enumeration was performed on days 1, 7, 14, 21 and 28. **b** ALP activity was measured spectrophotometrically in each cell culture well at days 4, 7, 14 and 21. *CTRL* control, *DIFF* differentiation, *FCS* fetal calf serum, *ns* not significant, *PLP* platelet lysate and plasma

To determine the rate of increase in cell number, cells were harvested and counted during the 28-day differentiation period (Fig. 1a). After plating, the number of PLP-grown MSCs increased 3.5-fold by day 7 of culture, and remained constant thereafter. The cells grown in FCS continued to proliferate throughout the experiment, resulting in significantly more cells on day 28 (FCS differentiated, 140,460 ± 17,793 cells/well; PLP differentiated, 39,000 ± 4258 cells/well; *p* < 0.01). The growth in MSC number of the control cultures paralleled that of the differentiating cultures.

### ALP activity in 2D cultures

Increase in ALP activity is known to accompany and influence the osteogenic differentiation. Spectrophotometric readings of ALP activity in PLP-grown cells peaked on day 4 (PLP differentiated, 0.27 ± 0.01 ng/well; FCS differentiated 0.13 ± 0.02 ng/well; *p* < 0.01), and declined thereafter (Fig. 1b). In FCS-grown cells, ALP activity rose by day 7, and remained elevated throughout the experiment (PLP differentiated versus FCS differentiated on day 14 and 21, *p* < 0.001).

#### Quantitative PCR analysis for osteogenic markers in 2D cultures

**Fig. 2:**
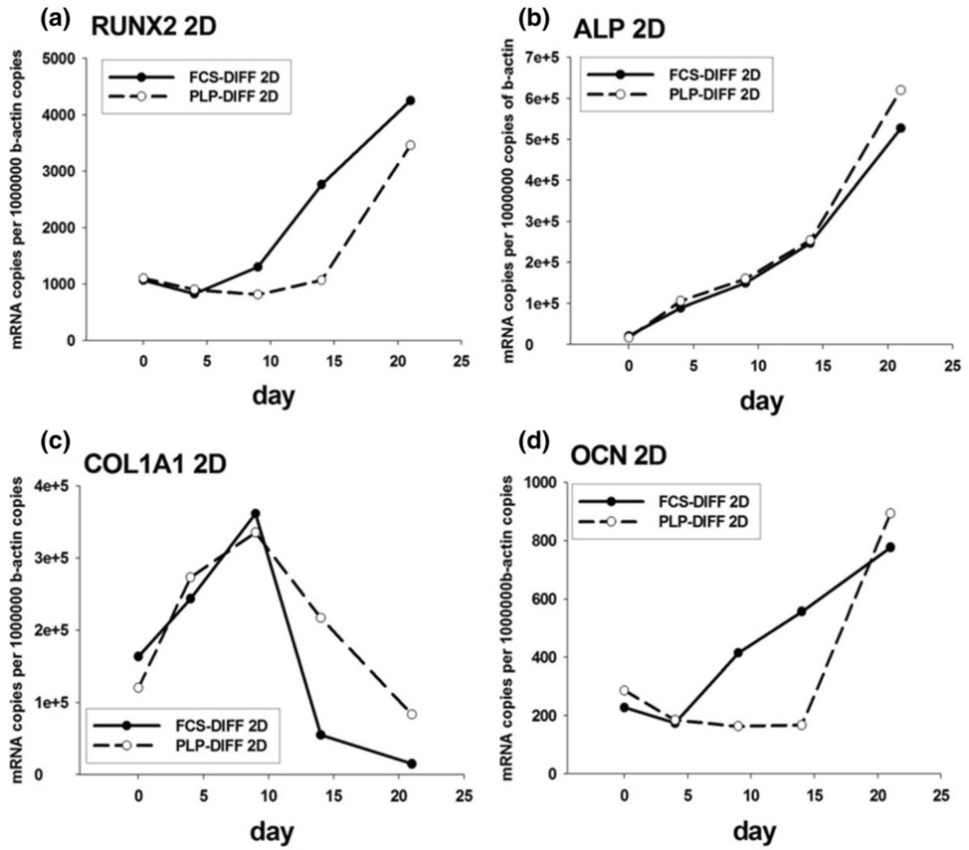
Quantitative RT-PCR in two-dimensional (2D) cell culture. The cells were cultured in 2D osteogenic medium up to 21 days. Total RNA was isolated and the expression of Runx2 (**a**), alkaline phosphatase (ALP) (**b**), collagen I α1 (Col1α1) (**c**), and osteocalcin (OCN) (**d**) was analyzed using quantitative RT-PCR. *DIFF* differentiation, *FCS* fetal calf serum, *PLP* platelet lysate and plasma

Osteogenic differentiation in 2D cultures was also examined by analyzing the expression of the Runx2, ALP, Col1α1 and OCN genes using quantitative RT-PCR. Increased Runx2 levels were found on days 9 and 14 in FCS but remained low in PLP cultures. In both conditions, Runx2 levels rose until day 21 (Fig. 2a). ALP mRNA levels increased steadily in both FCS and PLP cultures (Fig. 2b). Col1α1 levels increased steeply until day 9 after which the levels decreased in both FCS and PLP cultures (Fig. 2c). OCN mRNA rose until day 21 (Fig. 2d). In FCS cultures, elevated levels of OCN were seen from day 7 onwards, whereas in PLP cultures elevated OCN expression was seen only in late cultures.

### Detection of osteogenic matrices in 2D cultures

**Fig. 3:**
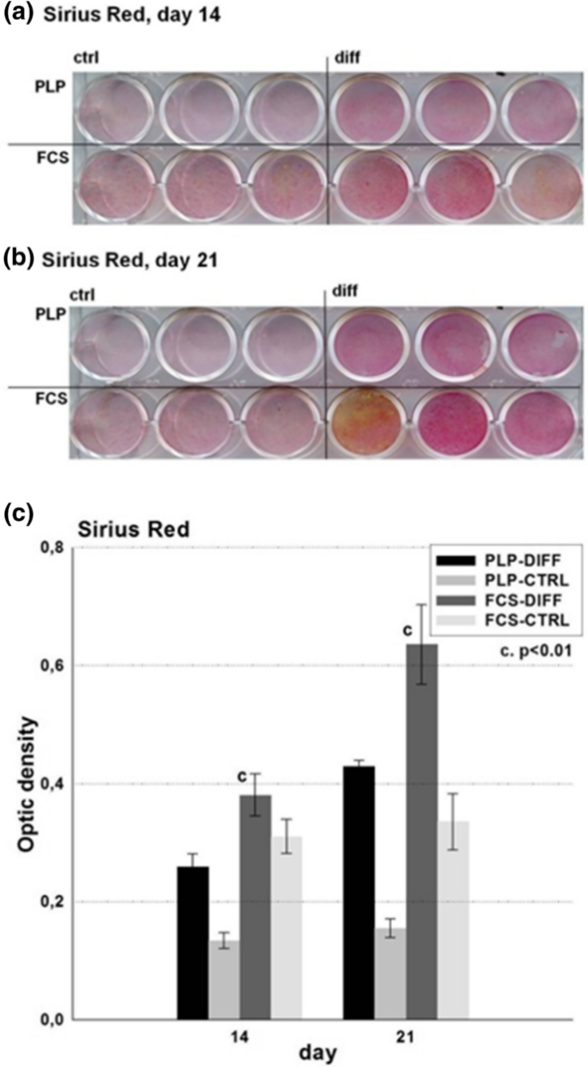
Detection of collagenous matrix in 2D cell culture. Cells were cultured in 2D osteogenic conditions up to 21 days in FCS and PLP culture media. At days **a** 14 and **b** 21, the cell cultures were fixed and stained with Sirius Red, after which they were dried and photographed. **c** Thereafter, they were turned into binary images and optical densities were measured. *CTRL* control, *DIFF* differentiation, *FCS* fetal calf serum, *PLP* platelet lysate and plasma

First, we analyzed whether the differentiating cells deposited a collagenous organic matrix, which is a prerequisite for bone formation. The matrix was detected using Sirius Red staining and quantified by measuring ODs. By day 14, both PLP and FCS cultures displayed low amounts of collagen matrix (Fig. 3a and c) which, using OD measurements, was stronger in FCS- than in PLP-grown cultures (*p* < 0.05). By day 21, the OD values had increased in all cultures, with the FCS cultures still showing the strongest staining (*p* < 0.05; Fig. 3b and  c). However, the OD values of PLP cultures were significantly higher (*p* < 0.001) than those of the control cultures.

**Fig. 4:**
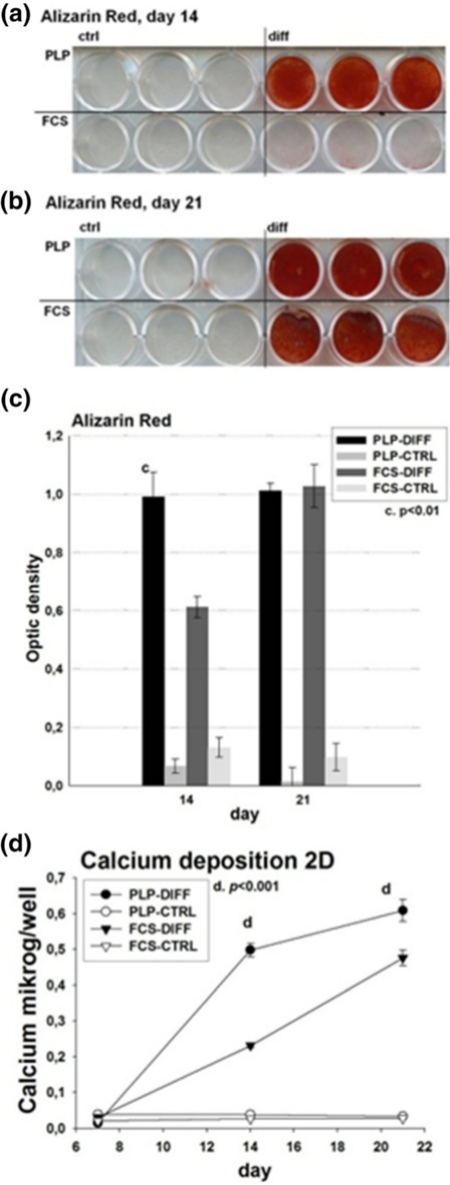
Detection of mineralized matrix and calcium deposition in two-dimensional (2D) cell culture. Cells were cultured in 2D osteogenic conditions up to 21 days in FCS and PLP culture media. On days **a** 14 and **b** 21, the cell cultures were fixed and stained with Alizarin Red, after which they were dried and photographed. **c** Thereafter, they were turned into binary images and optical densities were measured. **d** Deposited calcium was dissolved in HCl and quantified on days 7, 14 and 21 of culture using an o-cresolphthalein complexone reagent using a standard curve as reference. *CTRL* control, *DIFF* differentiation, *FCS* fetal calf serum, *PLP* platelet lysate and plasma

For detection of deposited mineralized matrix in the differentiating MSC cultures, the cultures were stained with Alizarin Red. By day 14 of differentiation, the PLP-grown cells displayed more intensive Alizarin Red staining than the FCS cultures (*p* < 0.01; Fig. 4a and c). By day 21, both PLP- and FCS-grown cells showed evidence of similarly rich mineralization (Fig. 4b and c). Quantitative data, acquired by measuring OD values, confirmed the visual impression (Fig. 4c).

### Calcium deposition in 2D

Because Alizarin Red staining is somewhat unspecific, we also quantified the calcium content of the deposited matrix (Fig. 4d). The deposited calcium in each well was measured spectrophotometrically. On day 7, the calcium levels were essentially equal in all samples, indicating that the deposition of calcium had not yet begun. On day 14, calcium levels had increased in the differentiating cultures, with the PLP culture showing the strongest response (PLP differentiated, 0.50 ± 0.02 μg/well; FCS differentiated, 0.23 ± 0.01 μg/well; *p* < 0.0001). On day 21, the values had increased further, being still highest in PLP cultures (PLP differentiated, 0.61 ± 0.03 μg/well; FCS differentiated, 0.48 ± 0.02 μg/well; *p* < 0.001).

### Osteogenic differentiation in 3D cultures

#### Increase in cell number during differentiation in 3D cultures

**Fig. 5:**
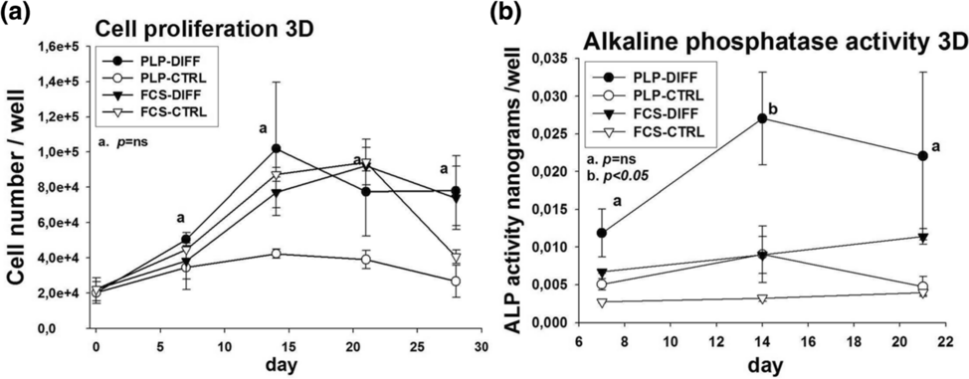
Cell proliferation and alkaline phosphatase (ALP) activity in three-dimensional (3D) cell culture. Cells (25 × 10^3^) were pipetted into 3D collagen sponge scaffolds and cultured up to 28 days. **a** Cells were harvested by dissolving the collagen scaffolds with collagenase B and counted thereafter at days 1, 7, 14, 21 and 28. **b** To measure ALP activity in 3D cultures, the collagen scaffolds were dissolved and cells were harvested. ALP activity was measured spectrophotometrically at 405 nm at days 4, 7, 14 and 21. *CTRL* control, *DIFF* differentiation, *FCS* fetal calf serum, *ns* not significant, *PLP* platelet lysate and plasma

To analyze the cell numbers in the differentiating 3D cultures, the matrices were dissolved with collagenase and the cells were recovered and counted (Fig. 5a). In PLP-differentiated cultures, the cell number peaked on day 14 and declined somewhat thereafter, while FCS-differentiated cultures peaked on day 21. Both differentiating cultures reached a similar final cell number by day 28 (FCS differentiated, 73,993 ± 17,862 cells/well; PLP differentiated, 78,047 ± 19,794 cells/well; nonsignificant), while the cell counts in the control cultures remained somewhat lower (26,640 ± 9041 cells/well in PLP control and 40,613 ± 3820 cells/well in FCS control).

### Alkaline phosphatase activity in 3D cultures

The pattern of ALP activity in 3D cultures differed substantially from that seen in 2D cultures. On day 7, the PLP-grown differentiating cells showed slightly higher levels of activity than the FCS-grown differentiating cells; however, this was without a statistically significant difference (PLP differentiated, 0.012 ± 0.003 ng/well; FCS differentiated, 0.0067 ± 0.0002 ng/well; nonsignificant). On day 14, the activity had further increased in PLP cultures, being significantly higher than in FCS cultures (PLP differentiated, 0.027 ± 0.006 ng/well; FCS differentiated, 0.0089 ± 0.002 ng/well; *p* < 0.05) with no further increase in activity in either condition thereafter (Fig. 5b).

#### Quantitative PCR analysis for osteogenic markers in 3D cultures

**Fig. 6:**
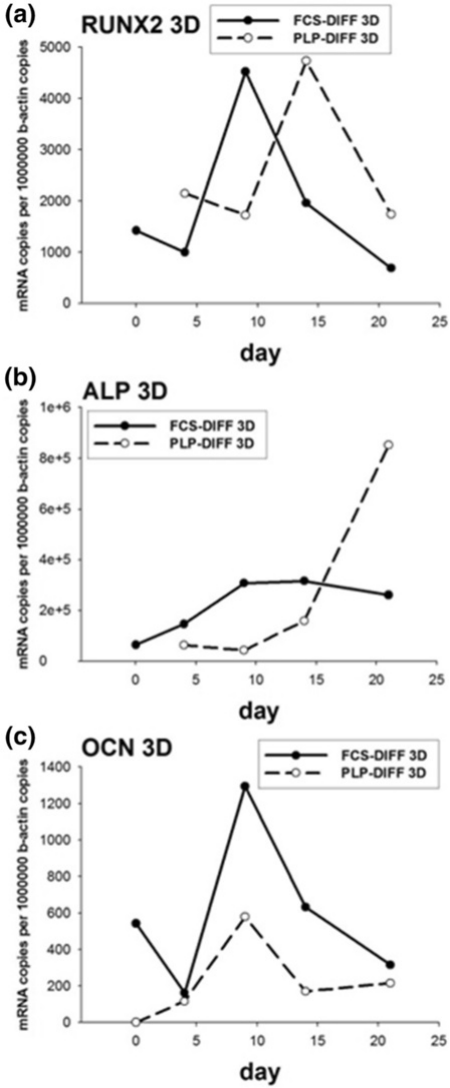
Quantitative RT-PCR in three-dimensional (3D) cell culture. The cells were cultured in 3D osteogenic conditions up to 21 days. Total RNA was isolated and the expression of Runx2 (**a**), alkaline phosphatase (ALP) (**b**), and osteocalcin (OCN) (**c**) was analyzed using quantitative RT-PCR. *DIFF* differentiation, *FCS* fetal calf serum, *PLP* platelet lysate and plasma

The mRNA profiles in 3D cultures also differed from those seen in the 2D cultures. Runx2 mRNA levels peaked on days 9 and 14 in FCS and PLP cultures, respectively (Fig. 6a). ALP mRNA levels rose relatively early in FCS cultures whereas a more marked rise in expression was detected in PLP cultures at 21 days (Fig. 6b). OCN levels peaked on day 9 in both FCS and PLP cultures, but were higher in FCS cultures (Fig. 6c).

### Detection of mineralized matrix and calcium deposition in 3D cultures

**Fig. 7:**
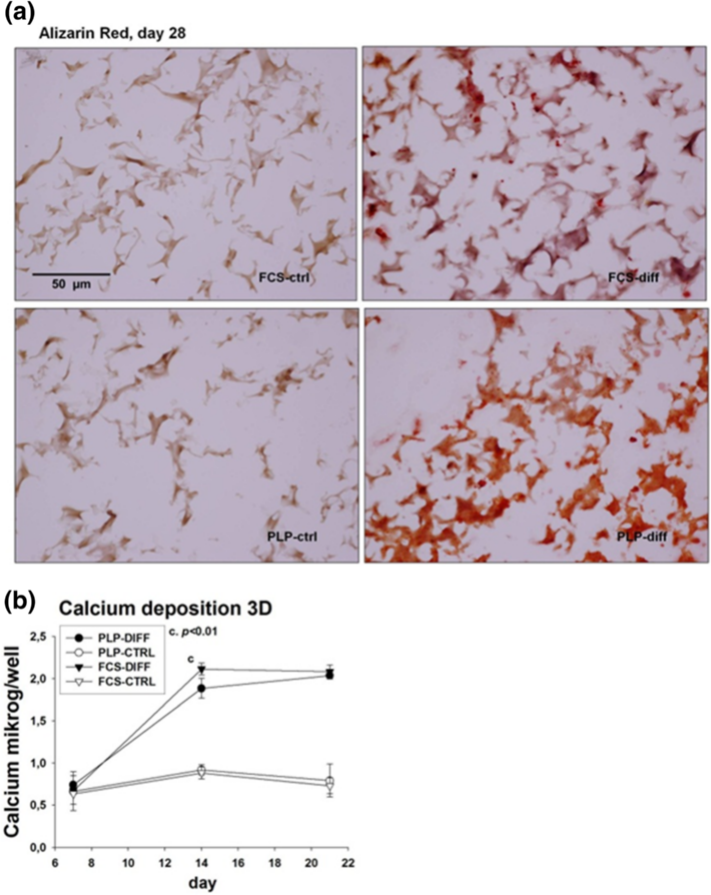
Detection of mineralized matrix and calcium deposition in three-dimensional (3D) cell culture. Cells were cultured in 3D matrices and fixed on day 28, mounted into Tissue-Tek and cut into 6 μm sections that were stained with Alizarin Red and photographed (**a**). Deposited calcium was quantified on days 7, 14 and 21 of culture (**b**). *DIFF* differentiation, *FCS* fetal calf serum, *PLP* platelet lysate and plasma

Sections of PLP and FCS 3D culture matrices stained with Alizarin Red displayed nodules of mineralized matrix on day 21 (not shown) and even more prominently on day 28 (Fig. 7a). No signs of mineralization were detected in the control cultures.

The presence of mineralized matrices in the 3D cultures was confirmed by analyzing the calcium content of the wells (Fig. 7b). Very little calcium was detected on day 7 in any of the culture conditions. On day 14, calcium levels rose sharply in both osteogenic FCS and PLP cultures, with FCS showing somewhat higher calcium levels (FCS differentiated, 2.11 ± 0.07 μg/well; PLP differentiated, 1.88 ± 0.12 μg/well; *p* < 0.01). Calcium levels remained relatively constant thereafter with no difference between PLP and FCS culture on day 28 (FCS differentiated, 2.08 ± 0.08 μg/well; PLP differentiated, 2.04 ± 0.04 μg/well; nonsignificant). The calcium levels remained low in control cultures without the osteogenic supplements.

## Discussion

MSCs are potential therapeutic agents for regenerative medicine if they can be expanded in sufficient amounts, grafted safely to the recipient, and induced to differentiate and demonstrate efficacy in vivo. The necessity of using animal-derived supplements for cell culture, carrying a risk of disease transmission and patient immunization, has been a major impediment for the application of cellular therapies in clinical practice. To circumvent this problem, we sought to substitute FCS with PLP in the MSC expansion and differentiation medium. The only animal-derived materials used for producing PLP cells were heparin and the collagen sponge. Both are approved for human medical use and pose little risk of transmission of xenogeneic infections.

For therapeutic use, the cells must retain their osteogenic potential and they must be able to produce bone matrix after implantation [26] or alternatively secrete osteogenic or immunomodulatory paracrine factors, which accelerate the natural bone healing.

A number of previous studies have shown that platelet extracts produced by various methods may be utilized to support osteogenic differentiation of MSCs [18, 19, 27–29]. In this report, we have studied the utility of PLP for expansion and differentiation of MSCs into osteoblasts in 2D and 3D cell cultures and compared their osteogenic potential with similar cells cultured using the commonly used cell culture constituent FCS. We used cells isolated from bone marrow aspirates of healthy donors and set up parallel cultures in PLP and FCS from the same donor. This was done to ensure that the cells in PLP would not be exposed to FCS at any time during culture. After expansion to the third passage, osteogenic inductors were added to the cells to analyze osteogenic differentiation. Parallel cultures are of importance because the osteogenic capacity can greatly vary between donors. The crucial parameters of osteogenic in vitro cultures are sufficient cell proliferation, ALP activity, expression of osteogenic markers and production of collagenous and mineralized matrices.

In our study, the cell number in 2D PLP cultures was smaller than in the FCS cultures throughout the in vitro differentiation, and cell number even decreased in later time points. One reason for this may be that PLP-grown cells seem to detach more easily from culture plates during the culture [30]. Recently we have shown that, in long-term 2D culture (more than 100 days), FCS-grown cells proliferate somewhat faster that MSCs grown in PLP [31]. In 3D sponges, FCS and PLP supported proliferation equally.

ALP expression and activity are prerequisites for osteoblast differentiation. ALP is also a ubiquitous marker expressed by all osteoblasts from the mature osteoprogenitor stage onwards [32]. In most of our experiments, ALP activity was higher in FCS-grown cells, however, with some variability between the donors. When mRNA levels of ALP were normalized to the level of the β-actin housekeeping gene, there was no difference in the ALP transcripts between PLP- and FCS-grown cells. Expression pattern of Runx2, another early osteogenic marker, was also similar in both FCS and PLP cultures.

Synthesis of a collagen matrix is required for the deposition of a mineralized matrix. Both processes are essential for treating bone defects. In order to analyze matrix production, the 2D cultures were stained with Sirius Red [33] and the expression of the main collagen type present in bone tissue, collagen I, was quantified with quantitative RT-PCR by measuring the Col1α1 gene. Sirius Red staining intensity was lower in PLP-grown cultures than in FCS cultures, which may reflect the lower cell number in PLP cultures. This is supported by PCR expression studies, which were normalized to the level of a housekeeping gene and revealed equal levels of mRNA of Col1α1 per cell in PLP and FCS cultures throughout the culture period.

In terms of mineralization, we found that PLP-cultured cells possess robust osteogenic features, reflected in the relatively high amount of mineralized matrix deposited at 14 day of the osteogenic differentiation. The level of mineralization, as evaluated by both Alizarin Red staining (Figs. 4a–c and 7a) and direct measurement of deposited calcium (Figs. 4d and 7b) was higher in PLP than in the traditional FCS cultures, in agreement with previous findings that mineralization starts earlier in PLP- than FCS-grown cells [19]. On the other hand, mRNA levels of OCN, a late osteogenic marker, was increased earlier in FCS than PLP cultures (Fig. 2d). OCN is expressed in postproliferative osteoblasts and appears concomitantly with mineralization but there is a heterogeneity between the donors [34]. Our results suggest that strong calcium deposition can be initiated in PLP cultures even though OCN levels are low in early cultures. OCN expression is strongly upregulated in a delayed fashion in PLP cultures. Compatible with this finding, Runx2, crucial for osteoblastogenesis and known to induce OCN expression [35, 36], correlated positively with the OCN levels in 2D cultures (Fig. 2a and d). These markers appeared before we observed a rise in calcium deposition on day 14. In therapeutic use, a maximal amount of well-mineralized matrix is likely to be of clinical benefit. In terms of mineral deposition, the PLP cultures appear to have an advantage over the FCS cultures, even though the expression of OCN seems to rise later than in FCS cultures.

Osteogenic differentiation in the 3D collagen sponge scaffold (Spongostan®) confirmed our findings in 2D cultures. Similar scaffolds have been previously used in osteogenic MSC cultures in a clinical trial treating congenital pseudoarthrosis in NF1 patients where healing of bone defects and bone formation were observed [7]. However, to our knowledge, there are no previous studies with PLP-grown MSCs in collagen sponge scaffolds.

Sufficient cell proliferation is mandatory for the therapeutic use of MSCs and it is needed both before and after the implantation of the cell graft. Unlike the 2D cultures, the collagen sponge scaffold seemed to support cell proliferation equally well in FCS and PLP cultures (Fig. 5a). Consistent with ample cell proliferation, ALP activity levels in 3D cultures were higher in PLP cultures than FCS cultures at day 14 (Fig. 5b). However, ALP mRNA increased earlier in FCS-grown than in PLP-grown MSCs (Fig. 6b).

We also visualized the osteogenic MSC cultures and their mineralization by staining the sponge culture sections with Alizarin Red. We observed mineralized nodules in both FCS and PLP cultures grown with osteoinductive supplements (Fig. 7a). This qualitative finding was confirmed by increased OCN mRNA copy numbers (Fig. 6d) and increased deposited calcium (Fig. 7b) in these cultures. Calcium levels were similar in PLP and FCS cultures. OCN mRNA peaked on day 9 in both FCS and PLP cultures preceding the increase in calcium level on day 14. The weakness of the current paper is that the presence of selected osteogenic marker proteins was not confirmed by, for example, western blotting, which should be addressed in future studies. Furthermore, this study was conducted in vitro, and the findings need to be evaluated also in in-vivo assays.

In summary, both PLP and FCS appear to support the osteogenic differentiation of MCSs, also when cultured in 3D collagen sponge scaffold. The osteogenic markers, however, show somewhat different temporal profiles during the culture period. As both FCS and PLP contain a complex mixture of different growth factors and other biologically active molecules (hormones and so forth) with varying and largely unknown concentrations, certain shifts in the differentiation time curve and in the expression of specific genes could be expected. Before executing these assays, we assumed that osteogenic differentiation would start earlier in 2D cultures because confluence was reached during the first few days of the culture. Surprisingly, Runx, one of the earliest markers of osteogenic differentiation peaked already on days 9–14 in 3D cultures, whereas 2D cultures did not show high Runx2 levels until towards the end of the culture period (Figs. 2a and 6a).

However, in choosing the best scaffold for repairing bone defects, other characteristics in addition to cell proliferation and differentiation are also needed, namely those related to safety, mechanical support and resilience. Collagen sponge scaffolds seem to support the MSC proliferation and differentiation during osteogenesis and have been widely used in surgery and are thus known to be safe in clinical practice. However, for orthopedic indications, they may not provide enough mechanical support for repair of large load bearing bone defects [7] and more rigid matrices are required. The data and methods from this study can be applied to further study such materials.

Although these experiments provided ample evidence for formation of mineralized matrix during MSC culture, higher quantities of the cells must be obtained for the therapeutic benefit. Longer cultures, increased cell density and addition of osteogenic growth factors may be needed to gain sufficient amounts of bone before the implantation [37]. Many other materials have already been used in implantation of bone grafts and may provide better mechanical support than collagen scaffolds used in the present experiments [27, 33, 38]. Finding the right vector for implantation, which supports sufficient cell proliferation both before and after the implantation, must be further examined.

For clinical use, the most crucial points are to find a culture method and a culture scaffold which together support both cell proliferation and osteogenic differentiation without exposing the recipient to the risk of infection and adverse host responses. Our findings suggest that MSCs grown and differentiated in PLP, without xenogeneic components, are a promising candidate for regenerative medicine in treating bone defects.

## Conclusions

Our data suggest that both FCS and PLP support MSC in vitro proliferation and osteogenic differentiation. As PLP is free of animal components and reduces the risk of xenogeneic infections, its use for bone repair in the clinic seems very promising. Osteogenic differentiation is a complicated multifactorial process, where not only soluble factors, but also the extracellular supporting conditions are at least as important. Accordingly, in our study MSCs differentiated in 2D and 3D conditions showed different sensitivities to being supported by PLP or FCS.

## Additional files

Additional file 1:
**Supplemental methods.** Methods used for analysis of MSC phenotypic markers and differentiation. (DOCX 20 kb) Additional file 2:
**Supplemental figures.** **Figure S1**: Analysis of MSC phenotypic markers by flow cytometry. **Figure S2**: Analysis of adipogenic and osteogenic differentiation potential of PLP- and FCS-grown cells (DOCX 2388 kb)

## Abbreviations

2D: Two-dimensional
3D: Three-dimensional
ALP: Alkaline phosphatase
Col1A1: Collagen I α1
FCS: Fetal calf serum
MSC: Mesenchymal stromal cell
NCBI: National Center for Biotechnology Information
OCN: Osteocalcin
OD: Optical density
PBS: Phosphate-buffered saline
PFA: Paraformaldehyde
PLP: Platelet lysate and plasma
pNPP: Para-nitrophenyl phosphate
RT-PCR: Real-time polymerase chain reaction.
