# Supplementary material for: Osteogenic differentiation of mesenchymal stromal cells in two-dimensional and three-dimensional cultures without animal serum
Source: Stem Cell Res Ther. 2015 Sep 7;6(1):167. doi: 10.1186/s13287-015-0162-6 (PMC4562352; doi:10.1186/s13287-015-0162-6)
Supplement: Additional file 1: — Supplemental methods. Methods used for analysis of MSC phenotypic markers and differentiation. (DOCX 20 kb) [file 13287_2015_162_MOESM1_ESM.docx]

### Manuscript 7176442615530552: Castren et al. Osteogenic differentiation of mesenchymal stromal cells in 2D and 3D cultures without animal serum

## Supplemental methods

### Analysis of phenotypic markers by flow cytometry

For immunophenotype analysis the cells were detached with TrypLE^TM^-express (Life Technologies) and washed with FACS buffer solution (0.3% BSA (Sigma-Aldrich) in PBS-2mM EDTA). The cells were labeled with Fluorescein isothiocyanate (FITC), phycoerythrin (PE) or allophycocyanin (APC)-conjugated antibodies against CD14, CD19, CD45, CD73, HLA-DR, (all from BD Pharmingen, San Diego, CA, USA), CD34 (Miltenyi Biotec GmbH, Gladbach, Germany), CD90 (StemCell Technologies Inc.) and CD105 (Abcam, Cambridge, UK). Appropriate FITC-, PE- and APC-conjugated isotype controls (all from BD Pharmingen) were used. The cells were analysed using a FACSAria flow cytometer and FACSDiva 5.0.3 (BD, San Jose, CA, USA) and FlowJo 7.6.1 softwares (TreeStar, Ashland, OR, USA).

### Differentiation assays

To assess adipogenic and osteogenic potential, passage two MSCs were plated onto 12-well plates (Nunc) at 3000 cells/cm^2^ and grown to confluency. For adipogenic differentiation the cells were changed into adipogenic induction medium for 2-3 days after which the cells were incubated in terminal adipogenic medium for 1-2 weeks. The induction medium and terminal differentiation medium consisted of the same adipogenic basal medium containing alpha-MEM Glutamax, 10% FBS, 20 mM HEPES, 100 U/ml penicillin, 100 µg/ml streptomycin (all from Life Technologies), 0.5 µg/ml insulin (Promocell, Heidelberg, Germany) and 0.1 mM indomethacin (Sigma-Aldrich, St Louis, USA). For induction medium, 0.5 mM 3-isobutyl-1-methylxanthine (IBMX), and 0.4 µg/ml dexamethasone (both from PromoCell) were added, and for terminal differentiation medium 3 µg/ml Ciglitazone (PromoCell) was added to basal medium. After differentiation the cells were fixed with 4% paraformaldehyde (PFA) and stained with Oil-Red-O or Sudan III (Sigma-Aldrich).

For osteogenic differentiation see ‘Methods’. After differentiation the cells were fixed with 4% PFA, the nuclei were counterstained with hematoxylin, and deposited calcium was detected with for von Kossa staining.
