# Supplementary material for: Osteogenic differentiation of mesenchymal stromal cells in two-dimensional and three-dimensional cultures without animal serum
Source: Stem Cell Res Ther. 2015 Sep 7;6(1):167. doi: 10.1186/s13287-015-0162-6 (PMC4562352; doi:10.1186/s13287-015-0162-6)

## Manuscript 7176442615530552: Castren et al. Osteogenic differentiation of mesenchymal stromal cells in 2D and 3D cultures without animal serum

## Supplemental figures

**Supplemental figure S1.** Analysis of MSC phenotypic markers by flow cytometry of cells grown in PLP (two columns on the left); and cells grown in FCS (two columns on the right). Each plot shows binding of isotype control (shaded graph) and specific mAb (open graph with solid line). The analyzed antigens are shown under each plot. Both PLP- and FCS-grown cells were negative for hematopoietic markers CD14, CD19, CD45 and HLA-DR. Both cell populations were positive for MSC markers CD73, CD90 and CD105.





**Supplemental figure S2**. Analysis of adipogenic (left column) and osteogenic (right column) differentiation potential of PLP- (top row) and FCS-grown (bottom row) cells.


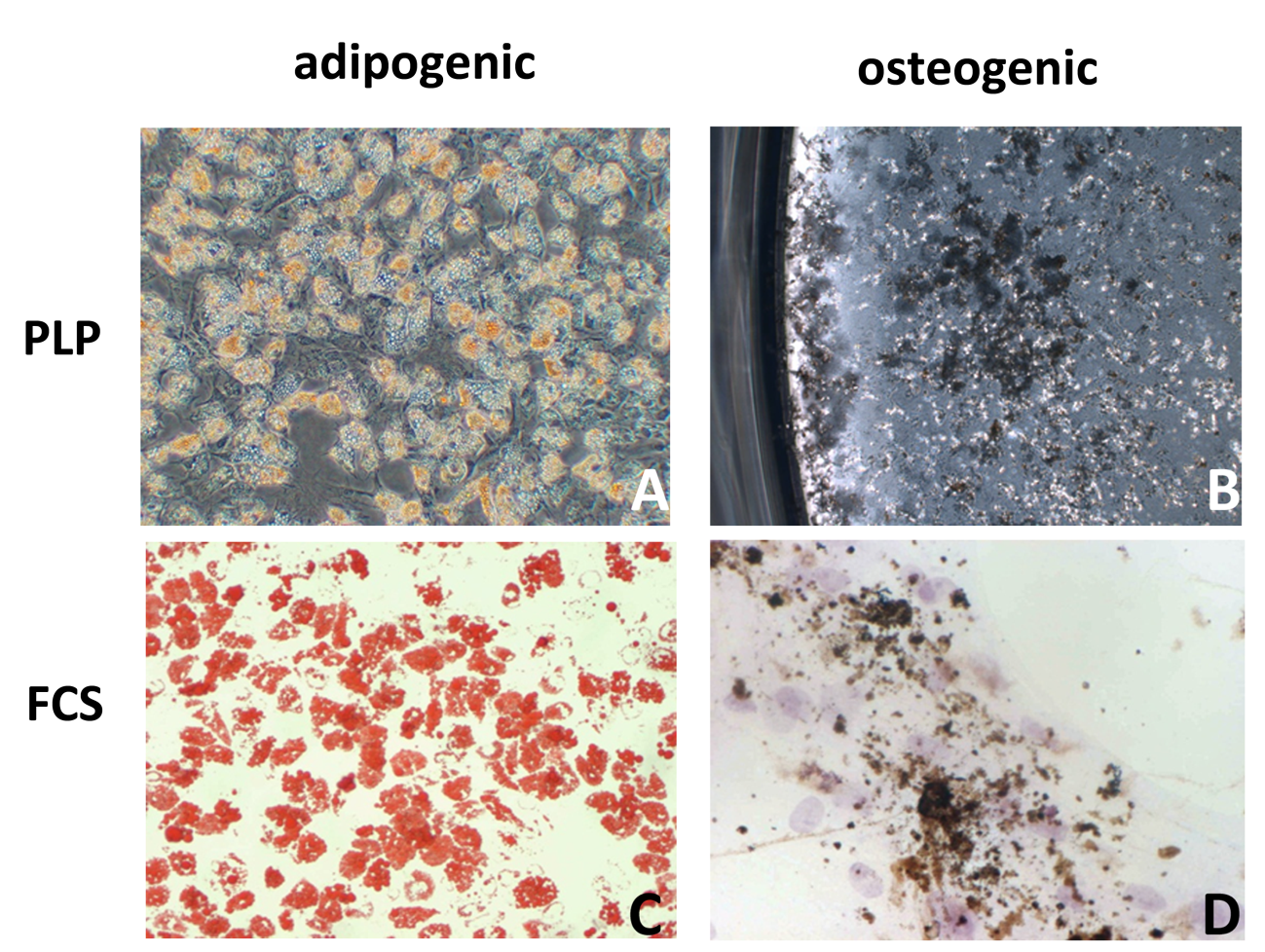

Supplement: Additional file 2: — Supplemental figures. Figure S1: Analysis of MSC phenotypic markers by flow cytometry. Figure S2: Analysis of adipogenic and osteogenic differentiation potential of PLP- and FCS-grown cells (DOCX 2388 kb) [file 13287_2015_162_MOESM2_ESM.docx]
